# Two Spx Regulators Modulate Stress Tolerance and Virulence in *Streptococcus suis* Serotype 2

**DOI:** 10.1371/journal.pone.0108197

**Published:** 2014-09-29

**Authors:** Chengkun Zheng, Jiali Xu, Jinquan Li, Luohong Hu, Jiandong Xia, Jingyan Fan, Weina Guo, Huanchun Chen, Weicheng Bei

**Affiliations:** 1 State Key Laboratory of Agricultural Microbiology, College of Veterinary Medicine, Huazhong Agricultural University, Wuhan, Hubei, China; 2 Key Laboratory of Development of Veterinary Diagnostic Products, Ministry of Agriculture, Huazhong Agricultural University, Wuhan, Hubei, China; 3 College of Food Science and Technology, Huazhong Agricultural University, Wuhan, Hubei, China; University of Rochester Medical Center, United States of America

## Abstract

*Streptococcus suis* serotype 2 is an important zoonotic pathogen causing severe infections in pigs and humans. The pathogenesis of *S. suis* 2 infections, however, is still poorly understood. Spx proteins are a group of global regulators involved in stress tolerance and virulence. In this study, we characterized two orthologs of the Spx regulator, SpxA1 and SpxA2 in *S. suis* 2. Two mutant strains (Δ*spxA1* and Δ*spxA2*) lacking the *spx* genes were constructed. The Δ*spxA1* and Δ*spxA2* mutants displayed different phenotypes. Δ*spxA1* exhibited impaired growth in the presence of hydrogen peroxide, while Δ*spxA2* exhibited impaired growth in the presence of SDS and NaCl. Both mutants were defective in medium lacking newborn bovine serum. Using a murine infection model, we demonstrated that the abilities of the mutant strains to colonize the tissues were significantly reduced compared to that of the wild-type strain. The mutant strains also showed a decreased level of survival in pig blood. Microarray analysis revealed a global regulatory role for SpxA1 and SpxA2. Furthermore, we demonstrated for the first time that Spx is involved in triggering the host inflammatory response. Collectively, our data suggest that SpxA1 and SpxA2 are global regulators that are implicated in stress tolerance and virulence in *S. suis* 2.

## Introduction

*Streptococcus suis* (*S. suis*) is an important zoonotic pathogen causing significant economic losses to the swine industry worldwide each year and is responsible for a variety of diseases in pigs, including meningitis, septicemia, arthritis, pneumonia, and even acute death [1]. Among the 33 serotypes (types 1 to 31, 33, and 1/2) identified on the basis of capsular polysaccharides, *S. suis* serotype 2 (*S. suis* 2) is considered to be the most virulent and the most frequently isolated serotype in association with diseases in most countries [2], [3]. *S. suis*, especially serotype 2, is also the causative agent of serious infections in humans in contact with infected pigs or pork-derived products and causes meningitis, septic shock, and permanent hearing loss [4]–[6]. Since the first reported case of *S. suis* infection in human in Denmark in 1968, more than 700 human cases worldwide have been recorded [7]. In 1998 and 2005, two large outbreaks of human *S. suis* 2 infection in China raised enormous public concerns because of the high pathogenicity of this microbe [4]. Recently, cases of human *S. suis* infection have been reported in Canada, the United States, Australia, New Zealand and Korea [8]–[10]. In addition, *S. suis* was identified as the leading cause of adult meningitis in Vietnam, the second most common cause in Thailand and the third most common cause of community-acquired bacterial meningitis in Hong Kong [9], [10].

The molecular pathogenesis of *S. suis* 2 infection remains poorly understood. During infection of the host, *S. suis* 2 requires a regulatory network to sense and respond to environmental signals [11]. A common regulatory mechanism used by *S. suis* for adaptation to environmental signals is two-component systems (TCSs). In *S. suis* 2, at least 15 TCSs have been predicted through bioinformatics analysis [12]. Among them, RevS [13], [14], SalK/SalR [15], CovR [16], CiaRH [17], Ihk/Irr [18], VirR/VirS [19] and NisK/NisR [20] have been described and shown to modulate virulence of *S. suis*. In addition to TCSs, *S. suis* also uses other regulators to respond to changing environments, several of which have been characterized. These include Zur [21], Rgg [11], LuxS [22], [23], CcpA [24], [25], PerR [26], AdcR and Fur [27]. To gain further insight into the global regulatory networks of *S. suis* 2, the role of other uncharacterized regulators should be investigated.

Spx proteins are a group of global transcriptional regulators that are highly conserved among low-GC-content Gram-positive bacteria [28]. Unlike most regulators that interact with promoter regions, Spx binds to the C-terminal domain (CTD) of the α-subunit of the RNA polymerase (RNAP) and alters the holoenzyme architecture [29]. By modifying the interactions between the RNA polymerase and specific promoter regions, Spx can activate or repress transcription [30]. To date, Spx regulators have been described in many bacterial species, including *Lactococcus lactis* [31], *Bacillus subtilis* [32]–[34], *Staphylococcus aureus* [35], [36], *Streptococcus pneumoniae* [30], *Streptococcus mutans* [37], *Streptococcus sanguinis* [38], and *Enterococcus faecalis* [39]. Regulation by Spx has been characterized in extensive detail in *B. subtilis*. Under conditions of disulfide stress, Spx regulates the expression of different subsets of genes, negatively or positively, by interacting with the CTD of the RNAP α subunit [32]. A crystal structure of the *B. subtilis* Spx protein, in complex with the α-CTD of the RNAP reveals interactions between Spx and the α-CTD, and suggests that subtle conformational changes may be important for the role of Spx in regulating organosulfur metabolism [33], [40]. A recent study identified 144 transcription units comprising 275 genes that were potentially under regulation by Spx in *B. subtilis* [34]. Using *B. subtilis* as a model organism, it was demonstrated that Spx is critical for the prevention of protein aggregate formation during thermotolerance [41]. Due to its important role in regulation, much attention has been paid to the role of Spx in organism pathogenesis. In *S. aureus*, Spx was shown to be a global effector impacting stress tolerance, biofilm formation and cell wall antibiotic resistance [35], [36]. In *S. mutans*, two Spx proteins were shown to modulate stress tolerance, survival and virulence [37]. SpxA1 was shown to be involved in X-state (competence) development in *S. pneumonia* [30], and involved in hydrogen peroxide production, stress tolerance and endocarditis virulence in *S. sanguinis* [38]. In addition, the Spx regulator was also shown to modulate stress responses and virulence in *E. faecalis* [39].

In this study, the roles of Spx regulators in *S. suis* 2 were explored. Two Spx proteins were identified: SpxA1 and SpxA2. Functional studies revealed that SpxA1 and SpxA2 play important roles in stress tolerance and virulence in *S. suis* 2. Global gene transcription profiles indicated that the two Spx proteins are global transcriptional regulators in *S. suis* 2.

## Materials and Methods

### Ethics statement

This study was performed in strict accordance with the recommendations in the Guide for the Care and Use of Laboratory Animals of Hubei Province, China. The protocol was approved by the Laboratory Animal Monitoring Committee of Huazhong Agricultural University. All efforts were made to minimize suffering.

### Bacterial strains, plasmids, and growth conditions

The bacterial strains and plasmids used in this study are listed in Table 1. *S. suis* 2 strains were grown in Tryptic Soy Broth (TSB) or plated on Tryptic Soy Agar (TSA; Difco Laboratories, Detroit, MI, USA) with 10% (vol/vol) newborn bovine serum at 37°C. *Escherichia coli* strain DH5α was grown in Luria broth (LB) liquid medium or on LB agar and used for plasmid construction and propagation. If required, spectinomycin (Sigma) was added to the growth media at the following concentrations, 100 µg/mL for *S. suis* 2 and 50 µg/mL for *E. coli*.

**Table 1:** Bacterial strains and plasmids used in this study.

Strain or plasmid	Relevant characteristics^a^	Source or reference
Strains		
SC19	Virulent strain isolated from the brain of a dead pig	Laboratory collection
Δ*spxA1*	*spxA1* deletion mutant of strain SC19	This study
Δ*spxA2*	*spxA2* deletion mutant of strain SC19	This study
DH5α	Cloning host for recombinant vector	TransGen
Plasmids		
pSET4s	*E. coli*-*S. suis* shuttle vector; spc^r^	42
pSET4s::*spxA1*	Recombinant vector with the pSET4s background,designed for knockout of *spxA1*	This study
pSET4s::*spxA2*	Recombinant vector with the pSET4s backgrounddesigned for knockout of *spxA2*	This study

a spc^r^, spectinomycin resistant.

### Construction of mutant strains

The *spxA1* and *spxA2* genes were deleted in the SC19 background by allelic exchange using the thermosensitive suicide vectors pSET4s as previously described [42]. Two flanking fragments (LA and RA) of an internal fragment of the *spxA1* gene (bases 1 to 342) were amplified from the *S. suis* 2 genome by PCR using primers listed in Table 2. After digestion with the appropriate restriction enzymes, the two PCR products were simultaneously cloned into pSET4s to generate a *spxA1* knockout vector, pSET4s::*spxA1*. The plasmid was introduced into SC19 competent cells by electroporation. After two steps of allelic exchange, spectinomycin-sensitive clones were selected and the presence of *spxA1* in the genome was detected by PCR using specific primers listed in Table 2. Reverse transcription PCR (RT-PCR) and DNA sequencing was performed to confirm the deletion. Δ*spxA2* was created in a similar manner.

**Table 2:** Primers used for PCR amplification.

Primer	Sequence (5′–3′)^a^	Product size (bp)	Target gene
A1U1	CCCCGTCGACAACTCTCGCTTATCATAGGCC	1046	Left arm of *spxA1*
A1U2	CGCCGGATCCCCGCGGAAGTATCGCA		
A1D1	CGCCGGATCCGTCTTCCATCCCCTCTAAAAAC	962	Right arm of *spxA1*
A1D2	CCGCGAGCTCGGTTTCACCGCTTTATCTACG		
A2U1	CCCCGTCGACTGGTTGTTGTTGACTCGGTT	927	Left arm of *spxA2*
A2U2	CGCCGGATCCGTAGTCACTCCTTTGCTACATTTTAT		
A2D1	CGCCGGATCCGATGTACAAAAGGACTCTTTTGG	1071	Right arm *of spxA2*
A2D2	CCGCGAGCTCCATTCTGCTGTTGCGTCTTC		
A1in1	CTTCGTTAAAACCAATCTGCAT	305	an internal fragment of *spxA1*
A1in2	GTCACCGAGTTGTACCAGTTGT		
A1out1	CTACCGCATCTGCTCCTTTT	804/462	a fragment containing *spxA1*
A1out2	CCTCTTGATGTTGGTTGGTATT		
A2in1	TCTTGTAAGAAAGCGAAAAATTG	292	an internal fragment of *spxA2*
A2in2	CATCTTCTTTATAGCCCACTTGA		
A2out1	GCAGATGGAGTTGAAGAAGTTG	1008/609	a fragment containing *spxA2*
A2out2	TAGCGAGGAATATAAGCAGGG		

a The underlined sequences are restriction sites.

### Stress challenges

To investigate the role of the two Spx regulators in stress responses, the wild-type (WT) and mutant strains were subjected to a variety of stress challenges (0.5 mM H_2_O_2_, 0.01% SDS, 1.5% NaCl, 0.3 mM diamide, 42°C, 30°C, pH 5.5, and medium lacking newborn bovine serum). Overnight cultures of the WT and mutant strains were diluted in fresh medium adjusted to each specific condition, and growth was evaluated by measuring the optical density at 600 nm (OD_600_) every hour.

### Mouse infections

100 female BALB/c mice (4–6 weeks old) were randomly divided into 10 groups with 10 mice per group. Groups I, II and III, which served as the high dose groups, were inoculated intraperitoneally with 7×10^8^ CFU in 200 µL PBS of the WT, Δ*spxA1* or Δ*spxA2* strain. Groups IV, V and VI, which served as the lower dose groups, were inoculated intraperitoneally with 3.5×10^8^ CFU in 200 µL PBS of each strain. Groups VII, VIII and IX were inoculated following the same protocol as Groups I, II and III, except that bacteria were heat-inactivated (10 min at 80°C) prior to inoculation. Mice in group X were injected with 200 µL PBS as the control group. Mice were monitored daily for 14 days to determine survival rates. Surviving animals were sacrificed on day 14 post-infection. After euthanization, brain samples were collected and prepared for histological examination. To examine bacterial burden, mice were injected intraperitoneally with 1×10^8^ CFU of each strain. At 24 h following injection, mice were euthanized for collection of blood, brain, and spleen samples. Brains and spleens were weighed and homogenized in 1 mL PBS. Homogenates were serially diluted and plated on TSA to determine the number of viable bacteria. Blood samples were directly diluted for plating.

### Histopathological studies

To examine differences in pathological changes, brain samples were collected from mice infected with the WT, Δ*spxA1* and Δ*spxA2* strains. Samples from the brain were fixed in 4% formaldehyde. After paraffin embedding, sections 4 µm thick were cut and stained with hematoxylin and eosin. Histology micrographs were obtained by light microscopy (Nikon, Tokyo, Japan).

### Competitive-infection assay

For competitive-infection assay, 200 µL of a 1∶1 Δ*spxA1*:WT or Δ*spxA2*:WT (5×10^7^ CFU) mixture was inoculated intraperitoneally into mice. The ratio in the inoculum was determined by plating a suspension of each strain prior to mixing. Mice were sacrificed to collect blood samples 18 h after inoculation, and blood samples were diluted for plating. The Δ*spxA1*:WT ratio in blood samples was determined by analyzing 70 colonies from each sample with colony PCR using primers A1out1 and A1out2, which yielded 462-bp and 804-bp PCR products for Δ*spxA1* and WT strains, respectively. The Δ*spxA2*:WT ratio was determined by the same method using primers A2out1 and A2out2, giving PCR products of 609-bp and 1008-bp for Δ*spxA2* and WT strains, respectively. The competitive index (CI) was calculated as the mutant:WT ratio in blood samples divided by the ratio in the inoculum.

### Measurement of Inflammatory Cytokines

To assess the differences in cytokine release trigged by the WT and mutant strains, a total of 36 infected and four uninfected mice were included for measurement of inflammatory cytokines. 36 mice were assigned randomly to three groups, and inoculated intraperitoneally with 2×10^8^ CFU of the WT, Δ*spxA1* or Δ*spxA2* strains. At 6, 9, 12, 15 h post-infection, three mice per group and one uninfected mouse were sacrificed for collection of blood samples. Serum samples were isolated and preserved at −80°C until analysis. Levels of IL-6 and TNF-α in serum were determined using commercially available enzyme-linked immunosorbent assay (ELISA) kits (Neobioscience, Beijing, China), following the manufacturer’s specifications.

### Bactericidal assays

Bactericidal assays were performed as previously described [43]–[45], with slight modifications. WT, Δ*spxA1* and Δ*spxA2* strains were harvested at the mid-exponential growth phase, washed three times, and diluted in PBS to yield 1×10^6^ CFU/mL. Subsequently, 900 µL heparinized blood from healthy pig (free of *S. suis*) was mixed with 100 µL bacterial suspension and incubated at 37°C for 3 h while rotating. The number of viable bacteria in each sample after 1 or 3 h and original inocula were determined by plating. The growth factor was defined as the ratio of CFU in each sample after 1 or 3 h incubation over the CFU in the corresponding inoculum.

### RNA isolation

*S. suis* strains were grown in TSB with 10% (vol/vol) newborn bovine serum to mid-exponential phase (OD_600_ = 0.6). Total RNA was isolated using an SV total RNA isolation system (Promega), according to the manufacturer’s recommended protocol. RNA concentrations and integrity were determined using an Agilent 2100 Bioanalyzer. The qualified RNA was then used for microarray analysis and qRT-PCR.

### Microarray analysis

DNA microarray analysis was performed using an Agilent custom-designed oligonucleotide microarray. Based upon the whole genome sequence of SC84 [46], specific 60-mer oligonucleotide probes were designed using eArray (https://earray.chem.agilent.com/earray/), to cover all annotated genes, with the exception of 10 genes for which it was not possible to design specific probes. The final coverage was 99.47% (1888/1898 genes). Probes were printed seven times on microarray slides. Two biological replicates of total RNA from WT strain and three from each mutant strain were amplified and labeled with Cy3-CTP using Low Input Quick Amp Labeling Kit, one-color (Agilent technologies, US), following the manufacturer’s instructions. Labeled cRNA was purified using the RNeasy mini kit (Qiagen). After fragmentation, microarray slides were hybridized with 600 ng Cy3-labeled cRNA. Hybridization was performed at 65°C for 17 h with rotation at 10 rpm. Microarray slides were washed and scanned by an Agilent Microarray Scanner (G2565CA). Those genes with greater than two-fold change ratios were regarded as differentially expressed genes. Microarray data has been deposited into the NCBI Gene Expression Omnibus (GEO) with accession number GSE56760.

### Quantitative Real-time PCR evaluation

A subset of genes was selected to confirm the accuracy of the microarray data by qRT-PCR with SYBR Green detection. The primers (Table S1) were designed according to the genomic sequence of SC84 [46]. First-strand cDNA was generated from total RNA using an Reverse Transcriptase kit (Toyobo, Japan). Quantitative PCR was conducted using the THUNDERBIRD SYBR qPCR Mix (Toyobo, Japan) according to the manufacturer’s instructions. Quantitative analysis was performed in triplicate with an ABI 7500 Fast Real-Time PCR system. 16S rRNA was used as a housekeeping control gene. The relative expression level was calculated using the comparative cycle threshold (2^−ΔΔCt^) formula normalized to the 16S rRNA level [47]. Student’s *t* test was performed to verify the significance of the real-time PCR quantifications.

### Statistical analysis

Statistical analysis was performed using GraphPad Prism 5 (San Diego, USA). Survival data were analyzed with the log-rank (Mantel-Cox) test. Differences in bacterial burdens were analyzed using the two-tailed Mann-Whitney test. The data in competitive-infection assay were analyzed using the two-tailed paired *t* test. The two-tailed unpaired *t* test was used to analyze the production of inflammatory cytokines in mice and bacterial survival in pig blood. *P*-values<0.05 were considered statistically significant.

## Results

### Identification of Spx homologues in *S. suis*

**Figure 1:**
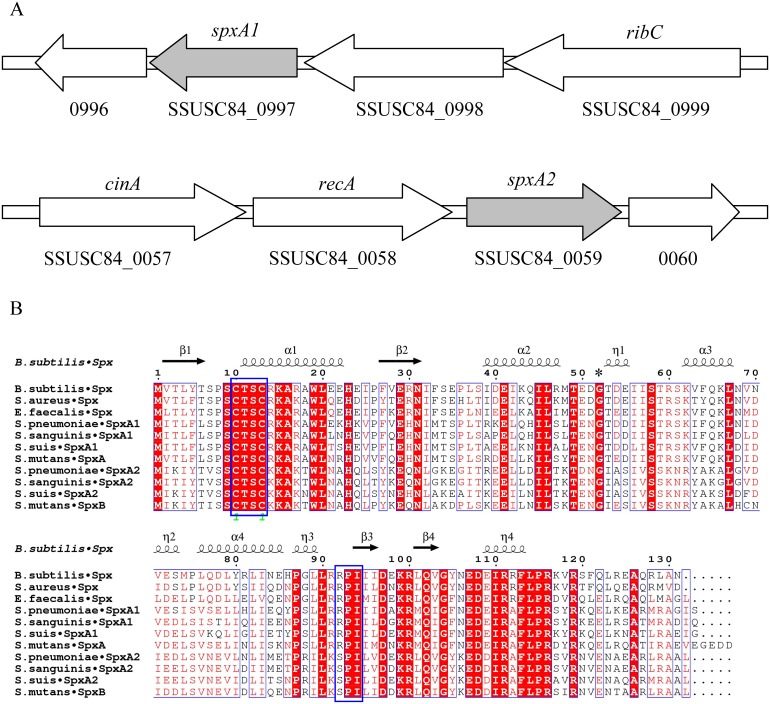
Identification of Spx regulators in *S. suis*. (A) Schematic representation of the *spx* locus in *S. suis*. The genes and the ORF number in *S. suis* SC84 genome are indicated. Arrows indicate the direction of transcription and do not represent the exact length. (B) Multiple sequence alignment of *S. suis* Spx proteins with related homologous proteins at the amino acid level. The multiple alignment was computed using ClustalW (http://www.genome.jp/tools/clustalw/), and the final image was generated using ESPript 3.0 (http://espript.ibcp.fr/ESPript/cgi-bin/ESPript.cgi). Identical residues are in white letters with red background, and similar residues are in red letters with white background. The secondary structure of Spx is shown on top. α: α-helix; β: β-sheet; T: β-turns/coils. The known crystal structure of *B. subtilis* Spx (Protein Data Bank entry 1Z3E) was used as a reference (33). The conserved CXXC and RPI motif discussed in the text are boxed, and the conserved Gly52 are labeled with an asterisk (*). The GenBank accession numbers are the following: *B. subtilis* Spx, NP_389032.1; *S. aureus* Spx, NP_374119.1; *E. faecalis* Spx, NP_816313.1; *S. pneumoniae* SpxA1, NP_358855.1; *S. sanguinis* SpxA1, YP_001034909.1; *S. suis* SpxA1, YP_003025001.1; *S. mutans* SpxA, NP_721528.1; *S. pneumoniae* SpxA2, NP_357767.1; *S. sanguinis* SpxA2, YP_001036156.1; *S. suis* SpxA2, YP_003024122.1; *S. mutans* SpxB, NP_722373.1.

A BlastP search against the proteins annotated in the genome of *S. suis* strain SC84 [46] was performed using the SpxA1 (locus tag *spr*1262) and SpxA2 (locus tag *spr*0173) proteins of *S. pneumoniae* strain R6 [48]. Two significant hits were identified, designated SpxA1 (locus tag SSUSC84_0997), and SpxA2 (locus tag SSUSC84_0059). The *spxA1* and *spxA2* genes of *S. suis* are located at opposite positions on the chromosome (Fig. 1A). SpxA1 consists of 133 amino acids with a predicted pI value of 6.74 while SpxA2 consists of 132 amino acids with a predicted pI value of 8.89. Pfam searches (http://pfam.janelia.org/) placed both proteins in the ArsC family. BlastP searches of protein database available from the National Center for Biotechnology Information confirmed that the two proteins are conserved across all sequenced *S. suis* strains. A multiple alignment was performed of *S. suis* SpxA1, SpxA2 and other Spx proteins, from either streptococci species (SpxA and SpxB from *S. mutans*, SpxA1 and SpxA2 from *S. pneumoniae* and *S. sanguinis*), or other species (Spx from *B. subtilis*, *S. aureus*, and *E. faecalis*). The results (Fig. 1B) showed that *S. suis* SpxA1 and SpxA2 possess two conserved residues/motifs: the amino terminal CXXC motif involved in redox state stress sensing by disulfide bond formation in *B. subtilis* [33], [49], and the Gly52 residue responsible for the interaction of *B. Subtilis* Spx with the RNA polymerase α-CTD [29], [33]. A carboxyl terminus RPI motif implicated in both modulating the reactivity of the CXXC motif and binding sulfate *in vivo* [33] is present in SpxA1, but is found as SPI in SpxA2. The high level of homology exhibited by these proteins suggests that SpxA1 and SpxA2 of *S. suis* may also share important functions similar to other Spx proteins.

### Microbiological characterization of *Δspx* strains

**Figure 2:**
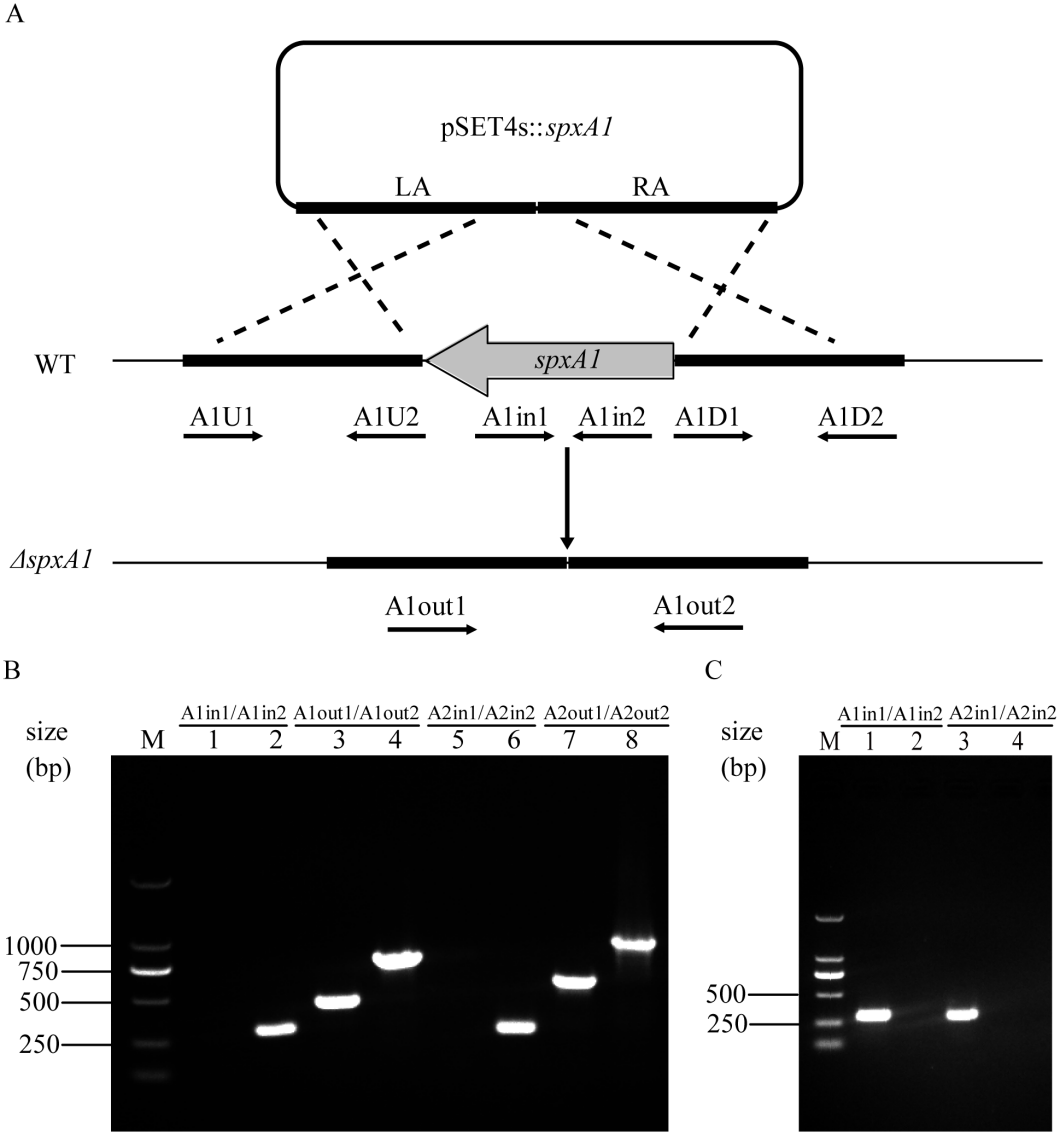
Construction and confirmation of the knockout mutant strains. (A) Strategy for deletion of *spxA1* in *S. suis* SC19 by homologous recombination. The plasmid pSET4s::*spxA1* is used for the *spxA1* gene knockout. LA and RA indicate the left and right arms of *spxA1*. (B) PCR confirmation of the mutant strains. The primer pairs used in the PCR analysis are indicated above the lanes. Genomic DNAs from the WT (lanes 2, 4, 6, and 8), Δ*spxA1* (lanes 1 and 3) and Δ*spxA2* strains (lanes 5 and 7), were used as templates. (C) RT-PCR identification of the mutant strains. Total RNAs were extracted from the WT, Δ*spxA1* and Δ*spxA2* strains. cDNAs generated from these RNA samples were subjected to RT-PCR analysis with primer pairs A1in1/A1in2 (for detection of *spxA1* gene transcripts) or A2in1/A2in2 (for detection of *spxA2* gene transcripts). The RT-PCR products were analyzed by electrophoresis on a 1% agarose gel (lanes 1 and 3, the WT strain; lanes 2, the Δ*spxA1* strain; lanes 4, the Δ*spxA2* strain).

To investigate the role of the Spx regulators in *S. suis* 2, in-frame deletion mutants of the two *spx* genes, designated Δ*spxA1* and Δ*spxA2* were constructed through homologous recombination (Fig. 2A). The mutation was confirmed by PCR detection (Fig. 2B), RT-PCR (Fig. 2C), and direct DNA sequencing of the mutation sites (data not shown). In both *S. mutans* UA159 and *S. sanguinis* SK36, a double mutant of the two *spx* genes is viable [37], [38]. In contrast, simultaneous inactivation of *spxA1* and *spxA2* in *S. pneumoniae* R6 is lethal [30]. We also failed to obtain the double mutant of the two *spx* genes in *S. suis* SC19.

**Figure 3:**
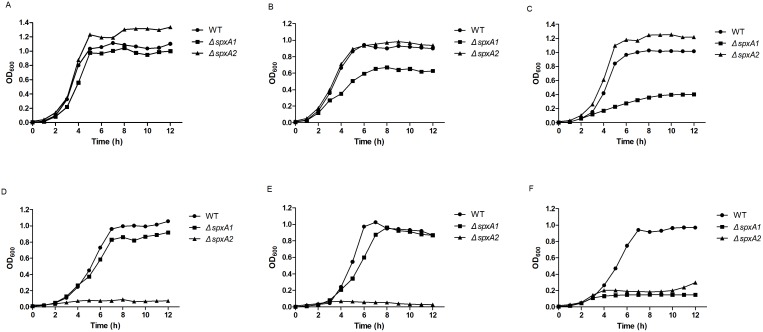
Growth curves of the WT, Δ*spxA1* and Δ*spxA2* strains. The WT (circles), Δ*spxA1* (squares) and Δ*spxA2* (triangles) strains were grown in TSB with 10% newborn bovine serum at 37°C under static conditions (A) or in a shaking incubator set to 180 rpm (B). For stress conditions, strains were inoculated in the presence of 0.5 mM H_2_O_2_ (C), 0.01% SDS (D), 1.5% NaCl (E) or in the absence of newborn bovine serum (F) and incubated at 37°C under static conditions. Growth was evaluated by measuring OD_600_. The curves shown are representative of a typical experiment performed three times.

The effects of deletion of the *spx* genes on the basic biological properties of *S. suis* were examined. In solid medium, Δ*spxA1* formed colonies of reduced size, while Δ*spxA2* showed no major difference from the WT strain. Deletion of the *spx* genes had no obvious effects on the haemolytic activity (unpublished observations). Cells grown to the exponential phase were observed by TEM (Fig. S1). Measurement of capsule thickness showed no obvious differences between the WT and mutant strains. The growth kinetics of *Δspx* strains were compared to those of the WT strain by measuring OD_600_ values. When grown in liquid culture at 37°C under static growth conditions, Δ*spxA2* produced fewer CFU (data not shown), but exhibited a higher OD_600_ value during the stationary phase than WT (Fig. 3A). In comparison, Δ*spxA1* demonstrated moderate reductions in OD_600_ value and CFU numbers (data not shown) during the stationary phase. In liquid culture with shaking at 180 rpm, Δ*spxA1* showed a significant defect in growth as compared with the WT, while the growth kinetics of Δ*spxA2* was almost identical to that of the WT strain (Fig. 3B).

### Role of SpxA1 and SpxA2 in stress tolerance

Spx regulators were found to play important roles in stress response in many species [35]–[39]. We, therefore, examined whether *S. suis* SpxA1 and SpxA2 were also involved in stress tolerance. The WT and mutant strains were cultured under a variety of stress conditions, and growth curves were compared with those of strains cultured under normal conditions (TSB with 10% newborn bovine serum at 37°C under static conditions). The results indicated that Δ*spxA1* is highly sensitive to hydrogen peroxide (Fig. 3C), while Δ*spxA2* is highly sensitive to SDS (Fig. 3D) and NaCl (Fig. 3E). Both mutants were defective when cultured in TSB in the absence of newborn bovine serum (Fig. 3F). However, no obvious growth difference was observed between strains cultured at 37°C and those cultured at a higher temperature (42°C, Fig. S2A), or a lower temperature (30°C, Fig. S2B). Furthermore, Spx proteins in *S. suis* were not associated with either acid tolerance (Fig. S2C) or resistance to diamide, a thiol-specific oxidant (Fig. S2D). These results strongly suggest that Spx proteins play a general role in stress response.

### Deletion of the *spx* genes affects *S. suis* virulence in mice

**Figure 4:**
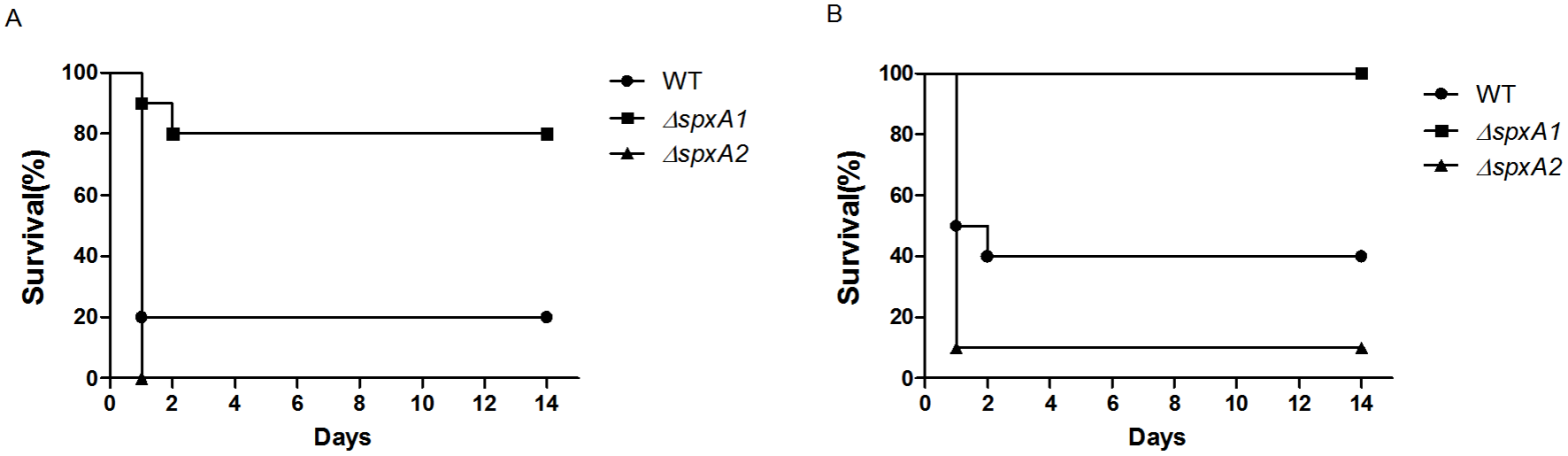
Survival curves of mice infected with *S. suis* strains. Groups of ten female BALB/c mice were inoculated intraperitoneally with the WT (circles), Δ*spxA1* (squares) and Δ*spxA2* (triangles) strains at a dose of 7.0×10^8^ CFU (A), or 3.5×10^8^ CFU (B). Survival was monitored over a 14 day period.

**Figure 5:**
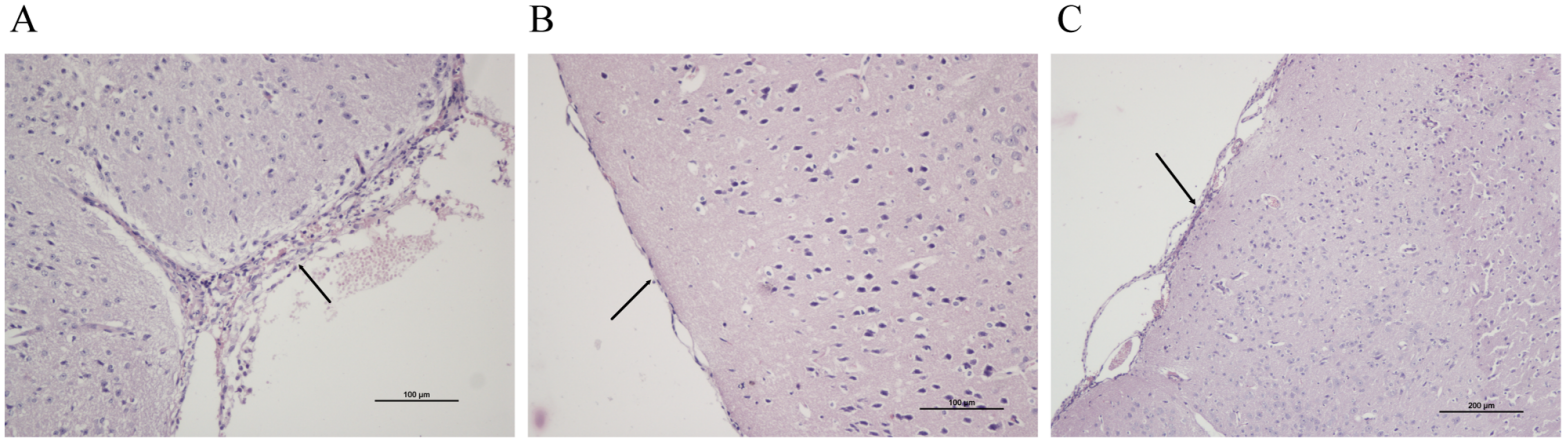
Pathological examination of brain tissues of mice infected with indicated *S. suis* strains. BALB/c mice were inoculated intraperitoneally with 3.5×10^8^ CFU of the WT, Δ*spxA1* and Δ*spxA2* strains. Brain samples were collected from surviving mice on day 14 post-infection and prepared for pathological examination. (A) The meninges of mice infected with the WT strain were severely thickened, infiltrated by macrophages and neutrophils. (B) No obvious change was displayed in the meninges of the mice infected with Δ*spxA1*. (C) The meninges of Δ*spxA2*-infected mice were mildly thickened.

Spx had been shown to modulate virulence in several Gram-positive bacterial species [37]–[39]. We investigated the role of *S. suis* SpxA1 and SpxA2 proteins in virulence using a murine infection model. In the higher inoculum dose groups, mice infected with the WT and Δ*spxA2* strains developed typical clinical symptoms of *S. suis* 2 infection, including rough coat hair, limping, lethargy, and swollen eyes. Mice infected with Δ*spxA1* mutant showed only mild symptoms. As shown in Figure 4A, 80% of the mice in the WT group and 100% of the mice in the Δ*spxA2* group died within 24 h. In contrast, only two mice in the Δ*spxA1* group died within 48 h. The survival rates were significantly lower in mice infected with the WT strain than in those infected with the Δ*spxA1* strain (P = 0.0059). No significant difference was observed between the WT-infected group and Δ*spxA2*-infected group (P = 0.1462). In the lower dose inoculum groups (Fig. 4B), mice infected with the WT and Δ*spxA2* strains also displayed typical clinical symptoms of *S. suis* 2 infection, with survival rates of 40% and 10%, respectively (P = 0.1003 for Δ*spxA2*). No mice in the Δ*spxA1* group developed clinical symptoms or died (P = 0.0040 for Δ*spxA1*). All mice inoculated with heat-inactivated bacteria or PBS remained healthy (data not shown). In addition, pathological examination showed that the meninges of the mice infected with the WT strain were severely thickened and a mass of macrophages and neutrophils could be observed. Similar pathological alterations occurred in the meninges of Δ*spxA2*-infected mice, but to a lesser degree, while the meninges of Δ*spxA1*-infected mice were similar to those of normal mice (Fig. 5). These findings indicate that the deletion of *spxA1* decreases the lethality of *S. suis* 2 in mice, and that the mortality was due to infection and not the toxic effects of bacterial components.

**Figure 6:**
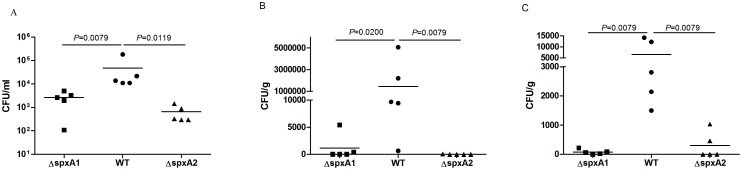
Colonization of the WT, Δ*spxA1* and Δ*spxA2* strains in various tissues of mice. Groups of five female BALB/c mice were inoculated intraperitoneally with 1.0×10^8^ CFU of the WT (circles), Δ*spxA1* (squares) and Δ*spxA2* (triangles) strains. Blood, brain and spleen were collected at 24 h post-infection. Bacterial burdens from blood (A), brain (B) and spleen (C) were examined. Statistical analyses were performed using the two-tailed Mann-Whitney test.

**Figure 7:**
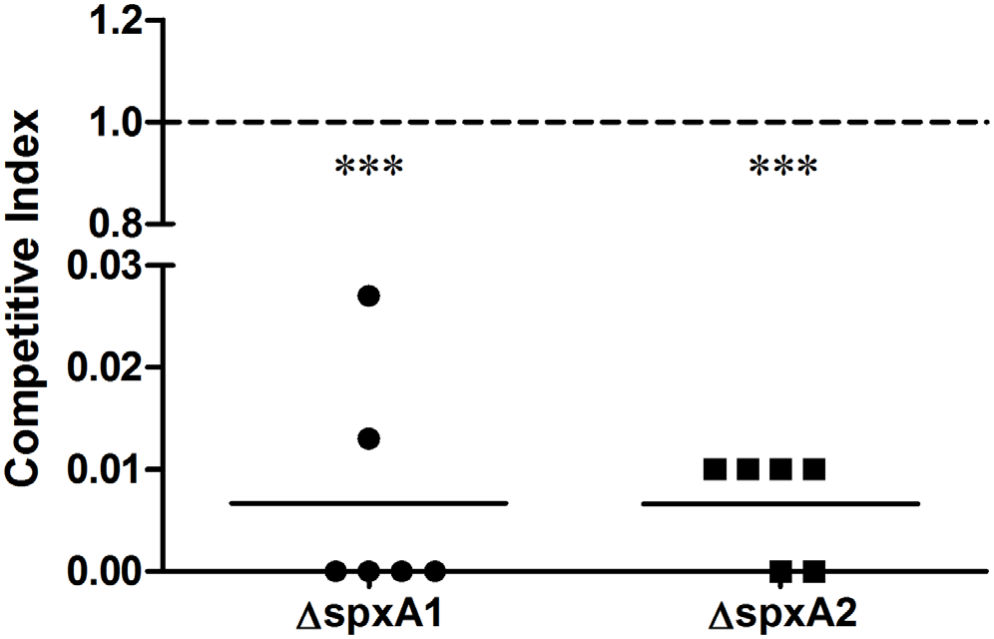
*In vivo* competitive index of Δ*spxA1* and Δ*spxA2* against the WT strain. Groups of six female BALB/c mice were inoculated intraperitoneally with a mixture of Δ*spxA1* and WT or Δ*spxA2* and WT at a ratio of 1∶1. At 18 h post-infection, blood samples were collected and plated. The Δ*spxA1*/WT and Δ*spxA2*/WT ratios were determined by analyzing 70 colonies of each sample with colony PCR. The competitive index was determined as the mutant:WT ratio in blood samples divided by the ratio in the inoculum. A CI value of 1 indicates equal competitiveness. Mean CI values from six mice were compared to 1 using the two-tailed paired *t* test to determine whether the difference in competitiveness is significant. ***P<0.0001.

To compare the abilities of the WT and mutant strains to establish infection, live bacterial cells from the three strains were examined in tissues of mice infected with sublethal doses of approximately 10^8^ CFU at 24 h post-infection. The number of bacterial cells of the WT strain recovered from the blood (Fig. 6A), brain (Fig. 6B), and spleen (Fig. 6C) was significantly higher than those from the Δ*spxA1* and Δ*spxA2* strains. Subsequently, competitive infection assays were performed to further evaluate the abilities of the WT and mutants strains to colonize the blood. Groups of six mice were inoculated intraperitoneally with a 1∶1 mixture of Δ*spxA1*:WT or Δ*spxA2*:WT. Bacterial cells recovered from the blood were analyzed by colony PCR to determine the CI. The results show that the CI values for Δ*spxA1* and *ΔspxA2 in vivo* were significantly less than 1 (Fig. 7), suggesting that both mutant strains had reduced abilities to colonize the blood.

### Induction of inflammatory cytokines in mice

**Figure 8:**
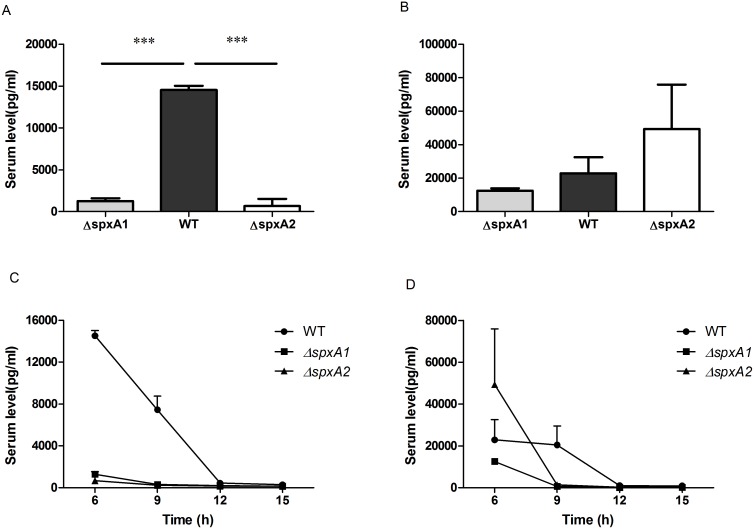
Production of inflammatory cytokines in mice. Serum levels of TNF-α (A) and IL-6 (B) in BLAB/c mice 6 h after infection with indicated *S. suis* strains at a dose of 2×10^8^ CFU. Time course of production of TNF-α (C) and IL-6 (D) in BALB/c mice infected with the WT (circles), Δ*spxA1* (squares) and Δ*spxA2* (triangles) strains. Data are expressed as mean levels ± standard deviation from three mice for each strain at each time point. Statistical analyses were performed using the two-tailed unpaired *t* test. ***P<0.0001.

To compare the capacity of *S. suis* strains to induce inflammatory cytokines, production of TNF-α and IL-6 was measured in the serum of infected mice. As shown in Figure 8A, the production of TNF-α, an important host mediator in the pathogenesis of septic shock [50], induced by either Δ*spxA1* or Δ*spxA2*, is significantly lower than that induced by the WT strain at 6 h post-infection. However, the production of IL-6, an important inducer of acute phase proteins [51], is clearly higher in Δ*spxA2* infected mice, and lower in Δ*spxA1* infected mice (Fig. 8B). To evaluate production of inflammatory cytokines over time after infection with *S. suis* strains, production of TNF-α and IL-6 was measured in serum of infected mice at four time points (6, 9, 12, 15 h after infection). As shown in Figure 8C and 8D, serum levels of TNF-α and IL-6 from the WT-infected mice decreased at 9 h post-infection, remained at high levels, and returned to basal levels at 12 h post-infection. The mutant strains Δ*spxA1* and Δ*spxA2* triggered a very low production of TNF-α. Additionally, serum levels of IL-6 from Δ*spxA1* and Δ*spxA2*-infected mice returned to basal levels at 9 h post-infection, approximately three hours earlier than the WT-infected mice.

### Deletion of the *spx* genes significantly attenuates survival of *S. suis* in pig blood

**Figure 9:**
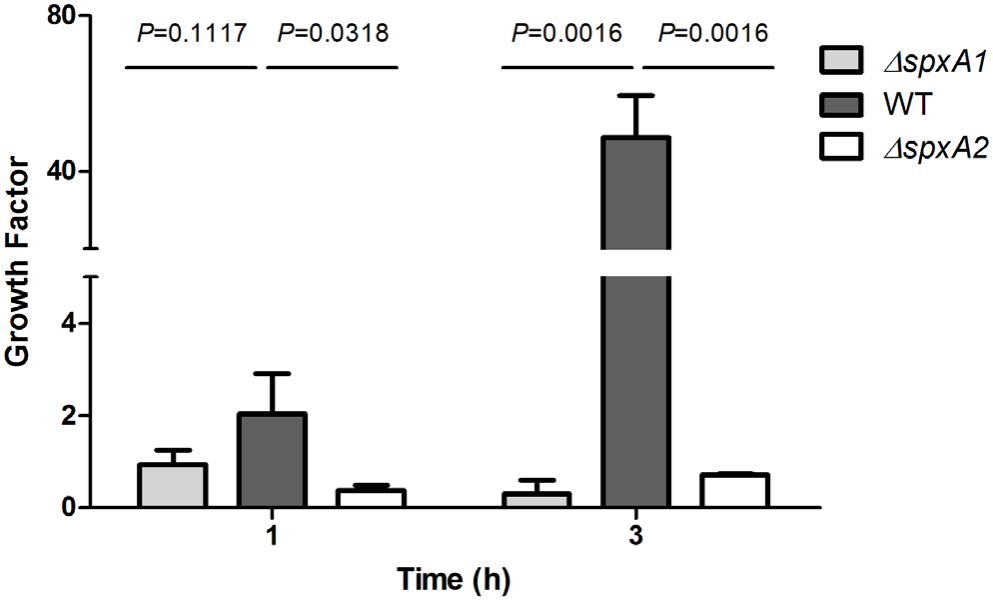
Growth factors of the WT, Δ*spxA1* and Δ*spxA2* strains in pig blood. Approximate 10^5^ CFU of the WT, Δ*spxA1* and Δ*spxA2* strains were incubated in heparinized pig blood and incubated for 3 h at 37°C with end-to-end rotation. Growth factor was defined as the ratio of CFU in each sample after 1 or 3 h incubation over the CFU in the corresponding inoculum. The results shown are the means ± standard deviations of three independent experiments. The *P* values were obtained using the two-tailed unpaired *t* test.

To determine whether deletion of the *spx* genes affects survival of *S. suis* in whole blood, we measured the ability of *S. suis* strains to grow in healthy nonimmune pig whole blood. After 1 h of incubation, the mean growth factors (ratio of *S. suis* CFU in cultures over inocula), of WT, Δ*spxA1* and Δ*spxA2* were 2.030±0.879, 0.937±0.306, and 0.373±0.117, respectively. After 3 h of incubation, the mean growth factors were 48.605±10.945, 0.303±0.287, and 0.710±0.030, respectively (Fig. 9). These results suggest the WT strain can evade immune components in blood and proliferate, while the mutants both showed decreased survival in blood.

### Microarray analysis reveals the global regulatory roles of SpxA1 and SpxA2

**Figure 10:**
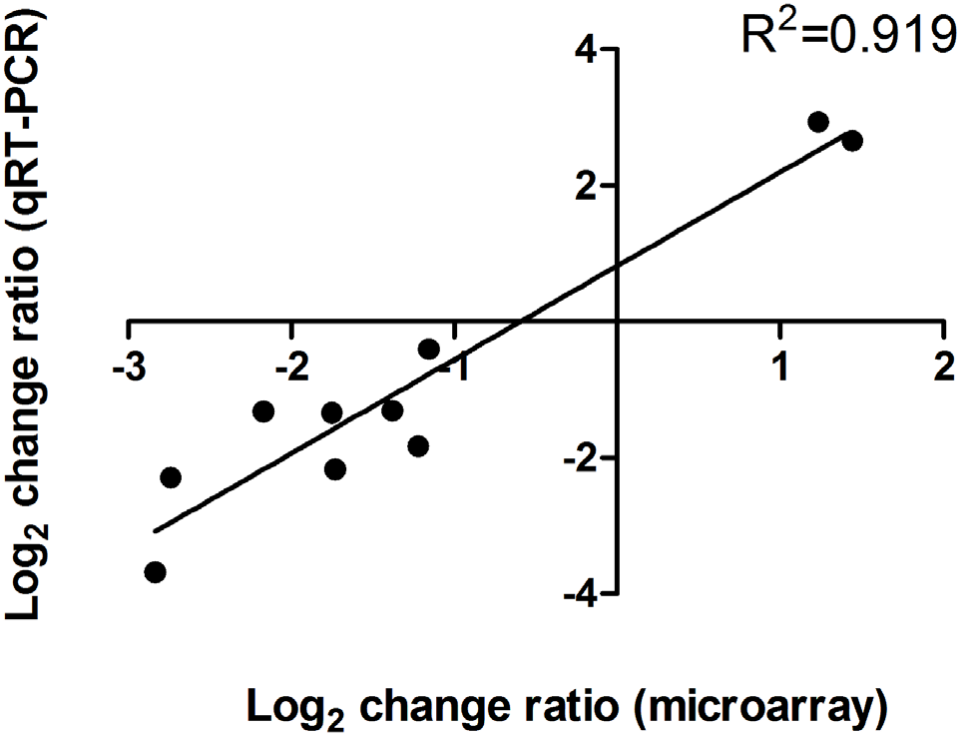
Correlation between DNA microarray data and qRT-PCR results. The relative transcriptional level of 10 selected genes determined by DNA microarray and qRT-PCR analyses were log_2_ transformed, and the values were plotted against each other to evaluate their correlation. The genes analysed by qRT-PCR are listed in Table S1.

To reveal the scope of the Spx regulation and explore the differences in the regulatory roles of SpxA1 and SpxA2, the global gene transcription profiles of the WT, Δ*spxA1* and Δ*spxA2* strains grown to mid-exponential phase were determined by DNA microarray analysis. As expected, the expression levels of flanking genes of *spx* were unaltered in the mutant strains, confirming that *spx* deletions don’t have a polar effect on the genes residing downstream of *spx*. A large number of genes were differentially expressed in each mutant strain compared to the WT strain (165 genes in Δ*spxA1* and 404 genes in Δ*spxA2*), revealing the global regulatory role of SpxA1 and SpxA2. Interestingly, there was minimal overlap between genes that were differentially expressed in Δ*spxA1* and Δ*spxA2* (only 15 genes expressed with the same trends in both strains), suggesting each Spx protein exerts its regulatory functions in an independent manner. These differentially expressed genes can be classified into several functional categories (Table S2), including information storage and processing, cellular processes and signaling, metabolism, and poorly characterized. A subset of 10 genes with varying expression levels was selected to confirm the accuracy of the microarray data by qRT-PCR. There was a strong positive correlation between the data obtained by the two methods (Fig. 10).

### Several genes involved in the oxidative stress response are positively regulated by SpxA1 and/or by SpxA2

The expression of several genes known to be involved in the oxidative stress response (*gor*, glutathione reductase; *nox*, NADH oxidase; *tpx*, putative thiol peroxidase; *sodA*, superoxide dismutase; *dpr*, Dps-like peroxide resistance protein) [37] were downregulated in Δ*spxA1* and/or in Δ*spxA2* (Table 3), suggesting these genes were positively regulated by SpxA1 and/or SpxA2. Downregulation of *nox*, *tpx* and *sodA* in Δ*spxA1* might be responsible at least in part for the impaired growth of Δ*spxA1* in the presence of hydrogen peroxide. In addition, *gor*, *tpx* and *dpr* were downregulated in Δ*spxA2*, suggesting that SpxA2 might also be involved in the oxidative stress response under other conditions.

**Table 3:** Expression ratios of genes involved in oxidative stress response and virulence in the mutant strains relative to the WT strain by microarray analysis^a^.

Locus_tag	Gene	Function	Δ*spxA1* strain	Δ*spxA2* strain
SSUSC84_0448	*gor*	glutathione reductase	ND	0.301 (0.222*)
SSUSC84_0648	*nox*	NADH oxidase	0.141 (0.078*)	ND
SSUSC84_1246	*tpx*	thiol peroxidase	0.150 (0.204*)	0.499
SSUSC84_1386	*sodA*	superoxide dismutase	0.222 (0.401*)	ND
SSUSC84_1526	*dpr*	Dps-like peroxide resistance protein	ND	0.449 (0.758*)
SSUSC84_1907	-	accessory pilus subunit	0.499	ND
SSUSC84_1908	-	accessory pilus subunit	0.309	0.444
SSUSC84_1385	*nudP*	surface-anchored 5′-nucleotidase	0.375	ND
SSUSC84_1224	*vicR*	response regulator protein	ND	0.384 (0.406*)
SSUSC84_1047	*endA*	competence associated endonuclease	ND	0.379

a The data in parentheses are qRT-PCR data. ND, no difference in expression levels. *P≤0.05.

### Expression of several proven or putative virulence-associated factors is altered in the mutant strains

Analysis of the microarray data revealed a change in expression of several genes involved in virulence (Table 3). Compared to the WT strain, the expression of genes encoding superoxide dismutase (*sodA*), accessory pilus subunit (SSUSC84_1907 and SSUSC84_1908) and Ectonucleotidase NudP (SSUSC84_1385) was downregulated in Δ*spxA1*. In addition, the microarray analyses also revealed downregulation of *vicR* (encoding response regulator protein of the VicRK TCS), *endA* (encoding competence associated endonuclease) and gene SSUSC84_1908 (encoding accessory pilus subunit) in Δ*spxA2*. These genes have been studied in *S. suis* or other streptococci species and shown to be implicated in the infection process [52]–[57]. Downregulation of virulence-associated factors might help to explain the phenotypes of the mutant strains in relation to the reduced abilities to colonize the tissues, and the decreased level of survival in pig blood.

## Discussion

Bacteria use global regulatory networks to sense and modify gene expression in response to changing environments. In many low-GC Gram-positive bacteria, Spx proteins are global transcriptional regulators that play a pivotal role in the regulation of stress tolerance and virulence [28], [37]–[39]. Although it has been described in a number of species, including the major streptococcal pathogens *S. pneumoniae* [30], *S. mutans* [37] and *S. sanguinis* [38], the role of Spx in *S. suis* has, so far, received little attention. Studies in *S. pneumoniae* [30], *S. mutans* [37] and *S. sanguinis* [38] uncovered two Spx proteins. In *S. pneumoniae*, simultaneous inactivation of *spxA1* and *spxA2* was lethal, but single-gene inactivation suggested that SpxA1 negatively regulates the X-state development by repressing transcription of the early competence operon *comCDE* [30]. In *S. mutans*, SpxA and SpxB were shown to modulate stress tolerance and were, for the first time, demonstrated to be required for virulence in Gram-positive pathogen [37]. In *S. sanguinis*, SpxA1 was shown to be involved in hydrogen peroxide production, stress tolerance and endocarditis virulence, while SpxA2 affected growth under normal conditions [38].

In the present study, we investigated the functional role of Spx in *S. suis* 2. Like some other streptococci [30], [37]–[38], two *spx* genes were discovered in the genome of *S. suis* 2, which were renamed *spxA1* and *spxA2*. Bioinformatics analysis showed that the Spx proteins were highly conserved not only among streptococci species, but also among other low-GC Gram-positive bacteria, suggesting that Spx regulators of *S. suis* 2 very likely function similarly to their orthologs in other bacteria, especially streptococci. To evaluate the role of Spx in *S. suis* 2, we constructed two mutants, Δ*spxA1* and Δ*spxA2*. Interestingly, we failed to obtain the double mutant of *spxA1* and *spxA2* in *S. suis* 2, which has similarly been noted in *S. pneumoniae* [30], suggesting their simultaneous inactivation is lethal. Although SpxA1 and SpxA2 share 45% identity at the amino acid level, independent deletion of *spxA1* and *spxA2* leads to different phenotypes, such as defective growth for Δ*spxA1* and normal growth for Δ*spxA2* in liquid culture with shaking at 180 rpm.

To investigate the involvement of SpxA1 and SpxA2 in stress tolerance, the WT, Δ*spxA1* and Δ*spxA2* strains were subjected to a variety of stress challenges. Our data show that Δ*spxA1* is highly sensitive to hydrogen peroxide, while Δ*spxA2* is highly sensitive to SDS and NaCl, and that both mutants are defective in medium lacking newborn bovine serum. The impaired growth of Δ*spxA1* under hydrogen peroxide stress, coupled with microarray analysis, indicates that SpxA1 plays an important role in the oxidative stress responses in *S. suis*. Similar result has also been reported for SpxA in *S. mutans* [37] and Spx in *E. faecalis* [39]. Although no oxidation-sensitive phenotype was observed in Δ*spxA2*, downregulation of several genes involved in oxidative stress suggests that SpxA2 might play a secondary role in control of oxidative stress or in regulation of the oxidative stress response under other conditions, such as anaerobic conditions. Unlike the staphylococcal *spx* mutant that was hypersensitive to high and low temperatures [35], no phenotype is associated with *spxA1* or *spxA2* deletion for heat or cold tolerance in *S. suis*, which is in agreement with *spxA1* inactivation in *S. pneumoniae* [30]. In addition, deletion of *spxA1* or *spxA2* did not affect the stress response to reduced pH. These findings are not surprising, as neither of the *spx* genes is included in the genes of *S. suis* 2 induced by temperature [58] or acidic stress [59]. Although Spx regulators have been reported to be involved in thiol-specific oxidative stress responses in many species [32], [35], [37], [39], mutant strains showed no significant differences in growth compared to the WT strain in the presence of diamide, a thiol oxidizing agent, similarly to results with Δ*spxA1* in *S. pneumoniae* [30].

To evaluate the role of SpxA1 and SpxA2 in the pathogenesis of *S. suis* 2, a murine infection model was used. Deletion of *spxA1* results in decreased lethality of *S. suis* 2 in mice while deletion of *spxA2* has no effect on lethality. In order to confirm that death of mice was due to infection and not to toxic effects of bacterial components, we performed infection of mice with heat-killed bacteria and observed no clinical symptoms. Pathological examination revealed that no obvious change was observed in the meninges of Δ*spxA1*-infected mice, while the meninges of Δ*spxA2*-infected mice suffered less damage than that of the WT-infected mice. This observation could be explained by subsequent colonization experiments, which showed that the number of bacterial cells of the mutant strains recovered from the brain was much less than that of the WT strain. Previous studies have shown that Spx contributes to colonization during infections, such as SpxA of *S. mutans* [37] and Spx of *E. faecalis* [39]. Our colonization analysis showed that recovered bacterial number of the mutant strains from the blood, brain, and spleen was significantly reduced. Competitive infection assay further revealed that the CI values for Δ*spxA1* and Δ*spxA2* in blood were significantly less than 1. These results suggest that both mutant strains reduce their abilities to colonize the tissues. It is surprising that Δ*spxA2* exhibits high lethality and reduced ability to colonize the tissues at the same time. Considering that the inflammatory response plays an important role in the pathogenesis of *S. suis* infection in mice [50], the effect of Spx regulators on the inflammatory response was investigated. The results show that serum levels of TNF-α in mice infected with mutant strains, either Δ*spxA1* or Δ*spxA2*, are significantly lower than in WT-infected mice. Although the difference is not significant, serum levels of IL-6 in mice infected with Δ*spxA2* are obviously higher than in WT-infected mice at 6 h post infection. Besides, Serum levels of TNF-α and IL-6 in mice infected with mutant strains return to basal levels at 9 h post infection, more quickly than in WT-infected mice. Previous study of group A streptococci showed that high levels of both TNF-α and IL-6 were inversely correlated with survival time in patients with sepsis [60]. We speculated that a higher dose of Δ*spxA2* induced excessive production of IL-6, which would damage the mice and lead to the high fatality rate. While mice were infected with a lower dose of Δ*spxA2* in colonization and competitive infection assays, low levels of IL-6 production would be beneficial for the mice to clear infection, thus lead to reduced Δ*spxA2* recovered from the tissues. To the best of our knowledge, this is the first description of Spx involvement in triggering of the host inflammatory response.

Dissemination via the bloodstream is considered to be an important step in the pathogenesis of *S. suis* 2 infection [61]. Bactericidal assays were further performed to examine survival of WT and mutant strains in pig blood. Both mutant strains show a significantly decreased survival ability in pig whole blood, indicating that SpxA1 and SpxA2 are involved in bacterial resistance to phagocytosis. The lower survival ability of mutant strains in blood might be partly responsible for their decreased abilities to colonize the tissues.

Considering the global regulatory role of Spx in *B. subtilis* [32], *S. aureus* [35] and *S. mutans* [37], microarray analysis of the WT and mutant strains was performed to identify genes under Spx regulation. The analysis revealed that transcription of a large number of genes is affected by SpxA1 and SpxA2, directly or indirectly. Of note, few genes are expressed with the same trends in both strains, suggesting that each Spx protein regulates gene expression independently, similarly to SpxA and SpxB in *S. mutans*. This might be the reason that we failed to obtain a mutant containing *spxA1* and *spxA2* deletions simultaneously, and that different phenotypes are observed in Δ*spxA1* and Δ*spxA2*. Downregulation of several genes involved in the oxidative stress response in mutants might be partly responsible for the phenotypes of defective growth under conditions of oxidative stress. It has been demonstrated that *vicR* inactivation could increase susceptibility to osmotic pressure in *S. pyogenes* [62]. The impaired growth under NaCl stress of Δ*spxA2* might be involved in the downregulation of *vicR* (Table 3). During the infection process, *S. suis* 2 may transform its metabolic mode to adapt to the new environment within the host [11]. Our data show that a large number of genes involved in metabolism are differently expressed in mutants, suggesting that Spx regulators in *S. suis* 2 play a critical role in regulation of genes involved in metabolism, which might not only be responsible for the defective growth of the mutant strains in culture without newborn bovine serum, but also facilitate the survival of *S. suis* 2 within the host. In addition, several proven or putative virulence-associated factors were downregulated in mutants, either Δ*spxA1* or Δ*spxA2*, helping to explain the reduced ability to colonize the tissues, and the decreased level of survival in pig blood. It has been shown that *S. suis*, especially its cell wall components, could induce both TNF-α and IL-6 production by murine macrophages [63]. Some genes involved in cell wall/membrane biogenesis were expressed differently in mutant strains (Table S2), which might be the reason that the two *spx* genes have an effect on induction of inflammatory response. Also, it is not surprising that Δ*spxA2* induced a higher production of IL-6, as the gene *pgdA*, which has been demonstrated to promote the production of IL-6 [64], was 2.16-fold upregulated in Δ*spxA2*.

In summary, bioinformatics, mutational and microarray analysis were used to identify and characterize two Spx regulators of *S. suis* 2. The present study clearly demonstrates that Spx regulators modulate stress tolerance in *S. suis* 2. Specifically, we show that Spx regulators globally modulate gene expression, especially genes involved in metabolism. We also demonstrate that the mutant strains show reduced abilities to colonize the tissues and decreased survival in pig blood. More importantly, this study demonstrates, for the first time, that Spx regulators are involved in triggering the host inflammatory response.

## Supporting Information

Figure S1 **Transmission electron micrographs of** ***S. suis*** **strains.** Bars, 200 nm. Bacteria were cultured in TSB containing 10% newborn bovine serum. Measurement of capsule thickness revealed that the thickness of capsules for the WT, Δ*spxA1* and Δ*spxA2* strains were 50.8±5.4 nm, 50.7±7.9 nm, and 51.7±6.0 nm, respectively.
(TIF)

Figure S2 **Growth of the WT,** **Δ*spxA1*** **and** **Δ*spxA2*** **strains under different stress conditions.** (A) Growth at 42°C. (B) Growth at 30°C. (C) Growth at pH 5.5. (D) Growth in the presence of 0.3 mM diamide. The curves shown are representative of a typical experiment performed three times.
(TIF)

Table S1 **Primers used for qRT-PCR analysis.**
(DOC)

Table S2 **Summary of genes classified by functional categories that were differentially expressed in** **Δ*spxA1*** **and** **Δ*spxA2*** **compared to the WT strain during mid-exponential growth as assessed by DNA microarray analysis.**
(DOC)
