# Supplementary figures and images for: Two Spx Regulators Modulate Stress Tolerance and Virulence in Streptococcus suis Serotype 2
Source: PLoS One. 2014 Sep 29;9(9):e108197. doi: 10.1371/journal.pone.0108197 (PMC4180751; doi:10.1371/journal.pone.0108197)

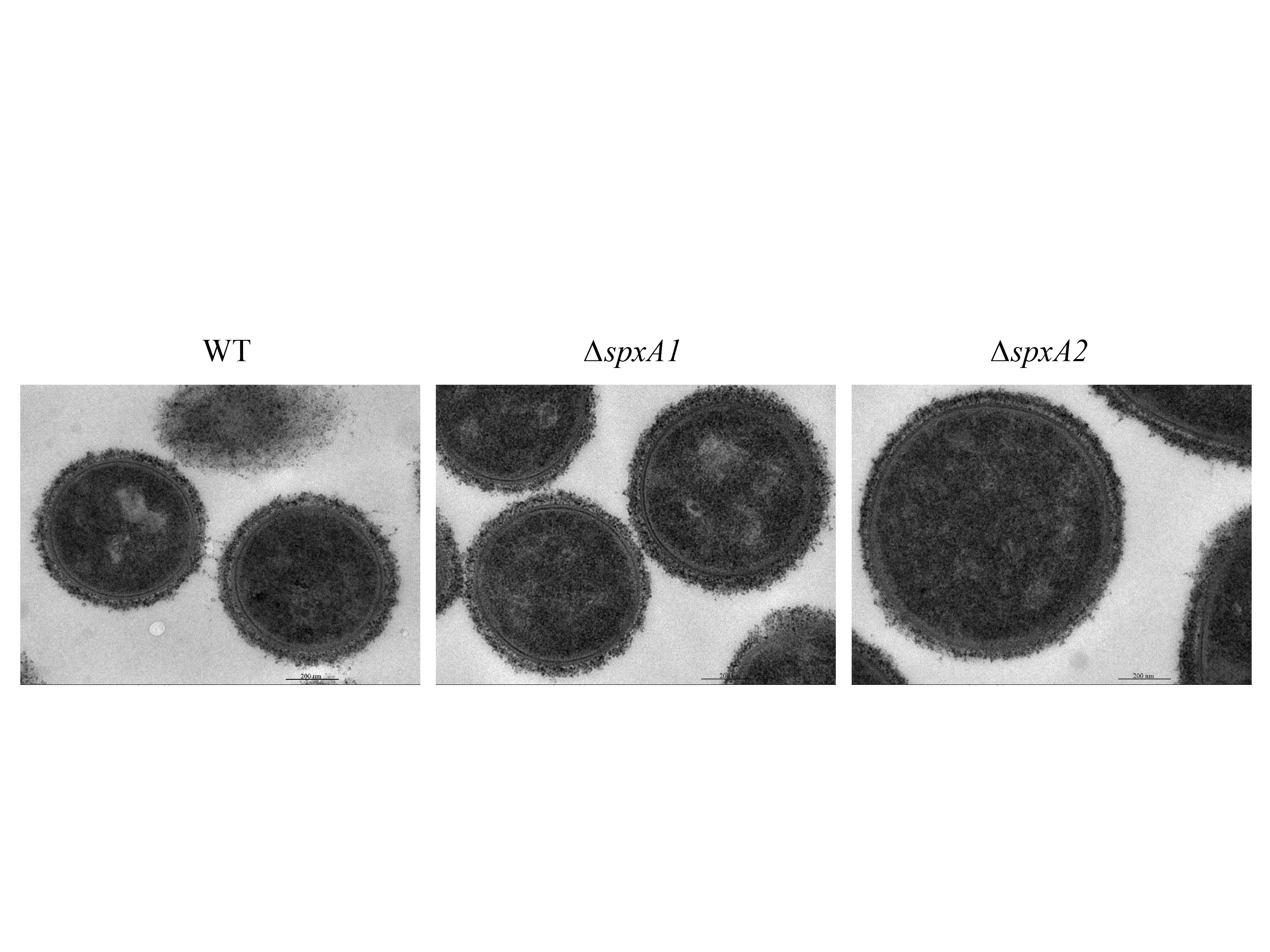

Supplement: Figure S1 — Transmission electron micrographs of S. suis strains. Bars, 200 nm. Bacteria were cultured in TSB containing 10% newborn bovine serum. Measurement of capsule thickness revealed that the thickness of capsules for the WT, ΔspxA1 and ΔspxA2 strains were 50.8±5.4 nm, 50.7±7.9 nm, and 51.7±6.0 nm, respectively. (TIF) [file pone.0108197.s001.tif]

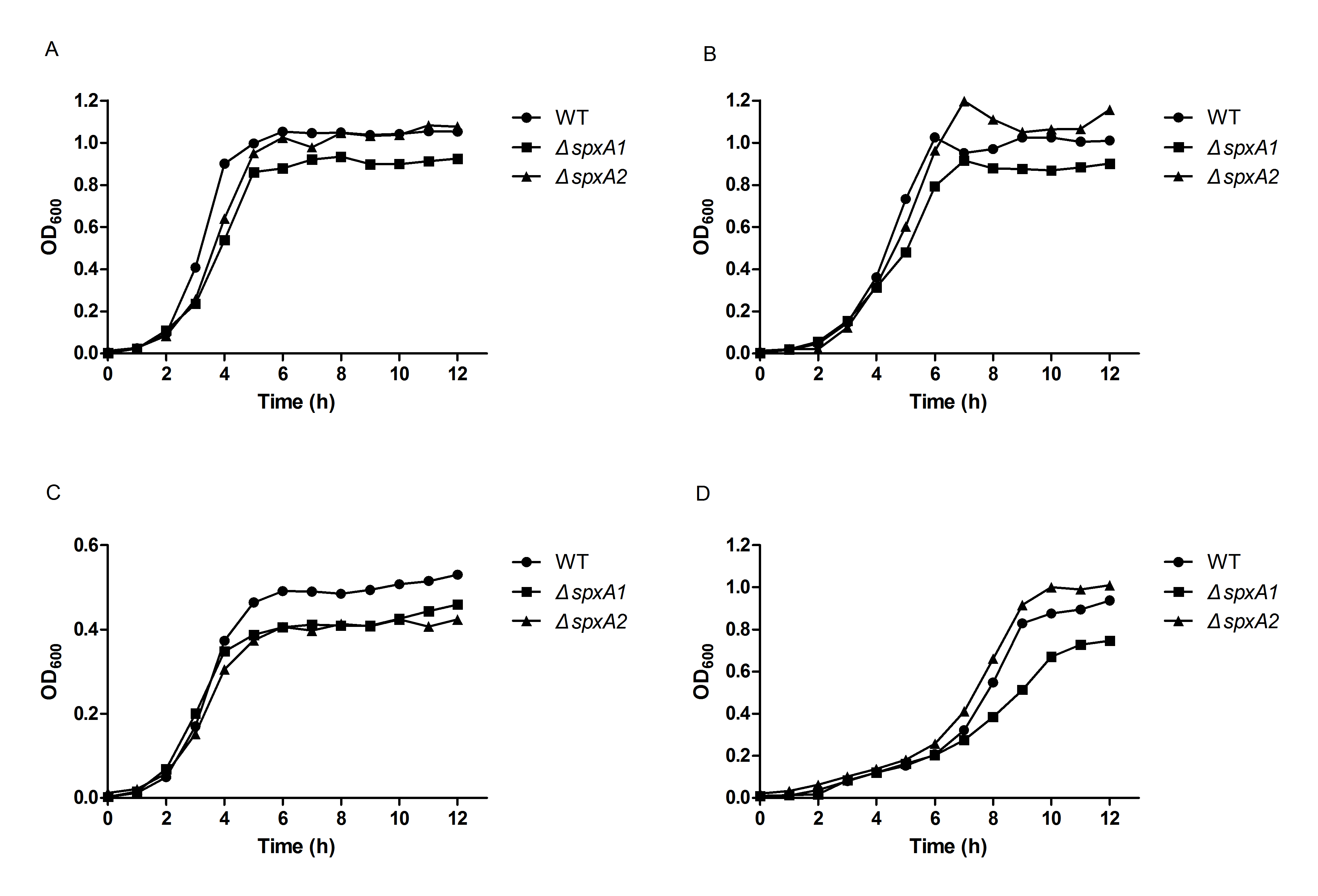

Supplement: Figure S2 — Growth of the WT, ΔspxA1 and ΔspxA2 strains under different stress conditions. (A) Growth at 42°C. (B) Growth at 30°C. (C) Growth at pH 5.5. (D) Growth in the presence of 0.3 mM diamide. The curves shown are representative of a typical experiment performed three times. (TIF) [file pone.0108197.s002.tif]
