# Supplementary material for: Two Spx Regulators Modulate Stress Tolerance and Virulence in Streptococcus suis Serotype 2
Source: PLoS One. 2014 Sep 29;9(9):e108197. doi: 10.1371/journal.pone.0108197 (PMC4180751; doi:10.1371/journal.pone.0108197)
Supplement: Table S1 — Primers used for qRT-PCR analysis. (DOC) [file pone.0108197.s003.doc]

**TABLE S1.** Primers used for qRT-PCR analysis.

| Primer | Sequence (5'-3') | Target gene |
| --- | --- | --- |
| 0648-F | ATGGTTATCTTGGCTGTTGGTTTCC | SSUSC84_0648 (*nox*) |
| 0648-R | GCCAGTAGCGTTGTCGTAGATTG |
| 1386-F | CCCTTCACCACGATAAGCACC | SSUSC84_1386 (*sodA*) |
| 1386-R | AAGGAATTTGCTCCACATCTGAC |
| 0584-F | TTCATCACCAAACACCCGC | SSUSC84_0584 (*comEC*) |
| 0584-R | ATCCTATCCACACCCCGACT |
| 1246-F | TCCGCAATTAAGAGTGGGGG | SSUSC84_1246 (*tpx*) |
| 1246-R | AAGCGACGGGTCTGGGTATC |
| 0448-F | GGCTGTGATTGGTGCTGGTTA | SSUSC84_0448 (*gor*) |
| 0448-R | GGAGTGGAAGGTTGGTACGTTG |
| 1224-F | TGCCAGAATTGGACGGACTAGAG | SSUSC84_1224 (*vicR*) |
| 1224-R | ACGAAGAAGAGCCTTAACACGC |
| 0555-F | GACCTTCGTGTCTTCTCTGTAACC | SSUSC84_0555 (*arcA*) |
| 0555-R | TGTATTTGAACCATCATTCCATTGC |
| 1526-F | GGGCACCATTTTCTACCCTTAAAG | SSUSC84_1526 (*dpr*) |
| 1526-R | CGGAACACCTCTACCACACG |
| 0827-F | AAGTGCCGCCAAATCCTTTC | SSUSC84_0827 (*tetM*) |
| 0827-R | ACAATCCGTCACATTCCAACC |
| 0583-F | AGTGGCTGGCTTGCTAATGG | SSUSC84_0583 (*comEA*) |
| 0583-R | TCTCTTCTGTACTGGTTTCCTCCG |
| 16s-F | ACTTGAGTGCAGAAGGGGAGAG | 16S rRNA |
| 16s-R | GCGTCAGTTACAGACCAGAGAGC |
