# Supplementary material for: Two Spx Regulators Modulate Stress Tolerance and Virulence in Streptococcus suis Serotype 2
Source: PLoS One. 2014 Sep 29;9(9):e108197. doi: 10.1371/journal.pone.0108197 (PMC4180751; doi:10.1371/journal.pone.0108197)
Supplement: Table S2 — Summary of genes classified by functional categories that were differentially expressed in ΔspxA1 and ΔspxA2 compared to the WT strain during mid-exponential growth as assessed by DNA microarray analysis. (DOC) [file pone.0108197.s004.doc]

**Table S2.** Summary of genes classified by functional categories that were differentially expressed in *∆spxA1* and *∆spxA2* compared to the WT strain during mid-exponential growth as assessed by DNA microarray analysis.

| Functional categories | Number of genes in *∆spxA1* | | Number of genes in *∆spxA2* | |
| --- | --- | --- | --- | --- |
|  | Upregulation | Downregulation | Upregulation | Downregulation |
| 1. Information storage and processing | 5 | 25 | 21 | 29 |
| 1.1 Translation |  | 14 | 9 | 5 |
| 1.2 Transcription | 4 | 8 | 8 | 10 |
| 1.3 Replication, recombination and repair | 1 | 3 | 4 | 14 |
| 2. Cellular processes and signaling | 11 | 14 | 37 | 38 |
| 2.1 Cell cycle control and mitosis |  |  | 7 |  |
| 2.2 Defense mechanisms |  | 1 | 9 | 4 |
| 2.3 Signal transduction mechanisms | 1 | 4 | 3 | 5 |
| 2.4 Cell wall/membrane biogenesis |  | 6 | 14 | 9 |
| 2.5 Cell motility | 3 |  |  | 2 |
| 2.6 Intracellular trafficking and secretion | 3 | 1 | 1 | 5 |
| 2.7 Posttranslational modification, protein turnover, chaperones | 4 | 2 | 3 | 13 |
| 3.Metabolism | 28 | 41 | 68 | 92 |
| 3.1 Energy production and conversion | 1 | 6 | 6 | 7 |
| 3.2 Carbohydrate | 10 | 16 | 14 | 22 |
| 3.3 Amino acid | 6 | 6 | 16 | 12 |
| 3.4 Nucleotide | 5 | 4 | 2 | 24 |
| 3.5 Coenzyme |  | 3 | 4 | 12 |
| 3.6 Lipid | 5 | 1 | 6 | 6 |
| 3.7 Inorganic ion | 1 | 4 | 18 | 7 |
| 3.8 Secondary metabolites biosynthesis, transport and catabolism |  | 1 | 2 | 2 |
| 4. Poorly characterized | 15 | 37 | 75 | 71 |
| 4.1 General function prediction only | 6 | 11 | 22 | 25 |
| 4.2 Function unknown | 2 | 7 | 11 | 13 |
| 4.3 Not in COGs | 7 | 19 | 42 | 33 |
| Total^a^ | 59 | 117 | 201 | 230 |

^a^ The genes assigned to more than one COG were counted repetitively.
